# Reported practices and difficulties for preventing emerging highly resistant bacteria in hemodialysis: the PHYDEL survey

**DOI:** 10.3205/dgkh000659

**Published:** 2026-06-23

**Authors:** Akel Azzi, Hélène Marini, Ludivine Boulet, Marion Lottin, Thomas Vermeulin, Hervé Vergnes, Stéphane Edet, Laurence Guet, Frank Le Roy, Véronique Merle

**Affiliations:** 1 Department of Infection Control, Rouen University Hospital, France; 2 Research group Dynamiques et Evènements des Soins et des Parcours, Rouen, France; 3 Comprehensive Cancer Centre Henri Becquerel, UNICANCER, Rouen, France; 4 CPIAS Occitanie, Toulouse, France; 5 REIN Registry, Agence de Biomédecine, Paris, France; 6 Nephrology and Hemodialysis Department, Rouen, France; 7 ANIDER Rouen Normandie, Rouen, France; 8 CPIAS Normandie, Rouen, France; 9 Department of Quality in Healthcare, Le Havre Hospital Group, Le Havre, France

**Keywords:** renal dialysis, carbapenemase-producing enterobacteriaceae, vancomycin-resistant enterococcus, infection control, screening

## Abstract

**Aim:**

Chronic hemodialysis patients are at risk of Vancomycin-resistant Enterocci (VRE) and carbapenemase-producing Enterobacteriaceae (CPE) carriage. France has released guidelines to prevent the spread of VRE-CPE carriage in hospitals. However, the hemodialysis setting is confronted with specific problems that are not always addressed by guidelines. Infection control practices regarding VRE-CPE transmission in the hemodialysis setting have not been described in the literature. The aim of our survey was to assess declared practices regarding VRE-CPE identification and management in French hemodialysis units (HDU).

**Methods:**

A cross-sectional survey was conducted in a random sample of 200 French HDU (excluding peritoneal dialysis and exclusive pediatric hemodialysis units) regarding their declared organization for identifying VRE-CPE carriers, the type and duration of precautions implemented, and reported difficulties. Data were collected via an e-mail questionnaire, and completed by a short telephone interview with an infection control nurse.

**Results:**

103 HDU agreed to participate. A minority of HDU had implemented a system to identify patients requiring screening or were able to keep track of VRE-CPE carriers and contacts via a computerized system. When managing VRE-CPE carriers, less than one-third of HDU employed caregivers, and strategies to discontinue precautions varied widely between HDU. HDU staff complained mainly about the lack of national specific guidelines for VRE-CPE in the hemodialysis setting, insufficient information regarding patients’ VRE-CPE status, and difficulties in convincing patients to accept screening.

**Conclusion:**

This survey highlights heterogeneous practices and routine difficulties in VPE-CPE patients’ management which would probably benefit from guidelines tailored to the hemodialysis setting.

## Zusammenfassung

**Zielsetzung:**

Bei Patienten mit chronischer Hämodialyse besteht das Risiko einer Übertragung von Vancomycin-resistenten Enterocci (VRE) und Carbapenemase-produzierenden Enterobacteriacae (CPE). Frankreich hat Richtlinien veröffentlicht, um die Ausbreitung vvon VRE-CPE in Krankenhäusern zu verhindern. Allerdings ist das Hämodialyse-Setting mit spezifischen Problemen konfrontiert, die nicht immer in Leitlinien behandelt werden. Infektionskontrollpraktiken im Hinblick auf die VRE-CPE-Übertragung im Hämodialysebereich wurden in der Literatur nicht beschrieben. Unser Ziel war es, die festgelegten Praktiken zur Identifizierung und Behandlung von VRE-CPE in französischen Hämodialyseeinheiten (HDE) zu bewerten.

**Methoden:**

In einer Zufallsstichprobe von 200 französischen HDE (ausgenommen Peritonealdialyse- und pädiatrische Hämodialyseeinheiten) wurde eine Querschnittsstudie hinsichtlich der Organisation zur Identifizierung von VRE-CPE-Trägern, der Art und Dauer der umgesetzten Vorsichtsmaßnahmen sowie der gemeldeten Schwierigkeiten durchgeführt. Die Daten wurden über einen E-Mail-Fragebogen erhoben, der durch ein kurzes Telefoninterview mit einer Krankenschwester für Infektionskontrolle ergänzt wurde.

**Ergebnisse:**

103 HDE erklärten sich zur Teilnahme bereit. Eine Minderheit der HDE hatte ein System zur Identifizierung von Patienten implementiert, die ein Screening benötigten, oder war in der Lage, VRE-CPE-Träger und -Kontakte über ein computergestütztes System zu verfolgen. Bei der Behandlung von VRE-CPE-Trägern setzte weniger als ein Drittel der HDE engagierte Pflegekräfte ein, und die Strategien zum Absetzen der Vorsichtsmaßnahmen variierten stark zwischen den HDEs. Das HDE -Personal beklagte sich hauptsächlich über das Fehlen nationaler spezifischer Richtlinien für VRE-CPE im Hämodialysebereich, unzureichende Informationen über den VRE-CPE-Status von Patienten’ und Schwierigkeiten, Patienten dazu zu bringen, das Screening zu akzeptieren.

**Schlussfolgerung:**

Die Umfrage beleuchtet heterogene Praktiken und Schwierigkeiten bei der Behandlung von VPE-CPE-Patienten, die wahrscheinlich von auf das Hämodialyse-Setting zugeschnittenen Leitlinien profitieren würden.

## Introduction

Chronic kidney disease (CKD) profoundly affects patients’ immune systems, leading to a heightened risk of infections. CKD induces a proinflammatory environment with elevated C-reactive protein and cytokines, impairing both innate and adaptive immunity. CKD-related metabolic disturbances and oxidative stress further exacerbate immune dysfunction, underscoring the need for tailored strategies to mitigate infectious risks in this vulnerable population [1]. Factors such as older age, chronic obstructive pulmonary disease, low serum albumin, immobility, and residence in care facilities increase the general risk of infections, while initiating dialysis with a catheter is strongly associated with a higher risk of bacteraemia [2]. Additionally, patients undergoing hemodialysis face an added risk of infection due to repeated vascular access and potential cross-transmission during dialysis sessions, where close proximity to other patients may contribute to the spread of infections [3]. Although survival rates for hemodialysis patients have improved significantly, hospitalization rates for infections such as bacteremia/septicemia and pneumonia/influenza have shown little change [2].

The global rise of carbapenemase-producing Enterobacteriaceae (CPE) and vancomycin-resistant Enterococci (VRE) is well-documented [4], [5]. In France, CPE and VRE are grouped under the term Emerging Highly Resistant Bacteria (eHRB), and measures have been recommended to control their spread, including strict contact precautions, screening of contacts, patients cohorting, and the organization of care during outbreaks [6]. Despite these interventions, the emergence of such bacteria remains a major challenge in many countries, including France [7], [8].

Hemodialysis patients are particularly vulnerable to the transmission of eHRB due to prolonged healthcare exposure, frequent use of indwelling catheters, and regular antibiotic treatments. These patients can also travel and undergo dialysis in different center, that may have a higher prevalence of eHRB, especially VRE, and therefore return contaminated after their trip [9], [10]. A meta-analysis gathering data from 100 healthcare facilities and 4,800 dialysis patients highlighted an overall 6.2% prevalence of VRE colonization among these patients [4]. A prospective survey demonstrated that 19% of those dialysis patients who remained hospitalized for four or more days became newly colonized with VRE [11]. In terms of CPE, hemodialysis has also been identified as a risk factor for colonization and infection [12], [13]. Improving adherence to infection prevention strategies is therefore crucial [3], [14]. 

The CDC has issued guidelines for managing multi-drug resistant organisms, recommending implementation of infection control measures in hemodialysis centers. including routine screening and isolation of high-risk patients, strict contact precautions for carriers, and effective antimicrobial stewardship to prevent resistance [15]. The Guidelines for Antimicrobial Resistance and Infection Control (AMRIC) in Ireland recommend that comprehensive measures including patient education about infection risks, regular screening for CPE, and implementation of strict hand hygiene protocols in hemodialysis settings [16]. The Canadian BC Renal Recommendations for screening and managing patients for CPE emphasize swabbing new and existing hemodialysis patients upon return from travel or discharge from units under investigation for CPE transmission, with follow-up swabs on days 1, 8, and 21. While awaiting test results, patients suspected of being CPE positive should be isolated, and contact precautions should be implemented, alongside rigorous cleaning protocols for HD equipment and patient areas [17]. In France, the 2019 High Council for Public Health (HCSP) recommendations for the prevention of eHRB in chronic dialysis units suggest conducting dialysis in dedicated rooms. If dedicated rooms are unavailable, it is recommended to group these patients together, with dedicated caregivers (i.e., cohorting) for their treatment sessions [18]. Additionally, regular screening of eHRB patients and their contacts should be conducted, with the frequency determined in collaboration between nephrologists and the local infection control team [18]. 

However, these recommendations can pose considerable logistical challenges for dialysis teams, especially in facilities with restricted space or limited resources, although there has been no formal assessment of these potential difficulties. Systematic evaluations of current eHRB prevention practices and barriers to adherence in French hemodialysis units has not been performed. 

Therefore, the aim of this study was to assess declared infection prevention control (IPC) practices regarding eHRB in a random sample of French hemodialysis units, including the protocol for identifying and managing eHRB carriers, the adherence to recommended infection control measures, and the declared barriers to adherence.

## Methods

### Study design and data collection

A cross-sectional study (the PHYDEL survey; **P**ratiques d’**H**ygiène en hémo**D**ialyse; **E**tat des **L**ieux, or, in English, overview of hygiene practices in hemodialysis), the methodology of which has been previously described [19], was conducted between March 2019 and December 2019 with the help of the REIN French national registry. This registry is a comprehensive database, funded by the French Ministry of Health, in charge of tracking epidemiological information on kidney disease and renal replacement therapies in France. The REIN registry lists all 1,236 French hemodialysis units. 

Out of 1,236 dialysis units listed by the REIN registry, those exclusively for pediatric or intensive care and exclusive peritoneal dialysis were excluded, leaving 1,189 potentially elegible units. A sample of 200 hemodialysis units was randomly selected from these 1,189 units. A questionnaire, based on the GREPHH survey [20] addressing various IPC measures, including hand hygiene, vaccination policy, antibiotic therapy policy, management of cross-transmission risks, vascular access, prevention of blood exposure accidents, and cleaning of hemodialysis unit’s premises, was drafted and tested. Specific questions about declared practices regarding eHRB management (Table 1 (Tab. 1)) – which are the subject of the present survey – were added to the baseline questionnaire. Participants were asked to describe the implementation of screening protocols in the hemodialysis unit for identifying at-risk patients and carriers, the establishment and utilization of computerized tracking systems for monitoring both patients carrying eHRB and their contacts, the regularity and methods of digestive screenings performed on these patients, and the specific difficulties encountered in effectively implementing these management strategies. As the survey was conducted in 2019, just before the release of new specific HCSP recommendations for hemodialysis [18], the questionnaire relied on the 2005 SF2H (French Society for Hospital Hygiene) recommendations for IPC in the hemodialysis setting [21]. Some of these recommendations were taken up in the 2019 HCSP guidelines [18].

The survey questionnaire was e-mailed to the 200 randomly selected hemodialysis units to be completed by a representative from each unit, along with the infection control team if possible. Hemodialysis units which did not reply within 15 days of the initial e-mail were e-mailed again once. 

Telephone interviews by a hygiene nurse supplemented the questionnaire to clarify responses, particularly regarding reported difficulties concerning eHRB patient management. 

### Analysis

Statistical analysis was carried out using Excel 11 (Microsoft) and OpenEpi 3.01 (Open Source Epidemiologic Statistics for Public Health, https://www.openepi.com/). Descriptive statistics were used to outline the characteristics of the dialysis units and summarize the findings for declared adherence to recommendations. Categorical variables were presented as percentages with corresponding 95% confidence intervals (95CI). 

In addition, free text comments regarding barriers for implementing SF2H recommendations were analyzed.

Results are reported according to Strengthening the Reporting of Observational Studies in Epidemiology (STROBE) guidelines.

### Ethical considerations

The survey received approval from the local institutional review board. No identifiable patient information was gathered, so there was no need for approval from the French Electronic Data Protection Authority. The authors committed to strictly protect the confidentiality of the hemodialysis units.

## Results

**Figure 1:**
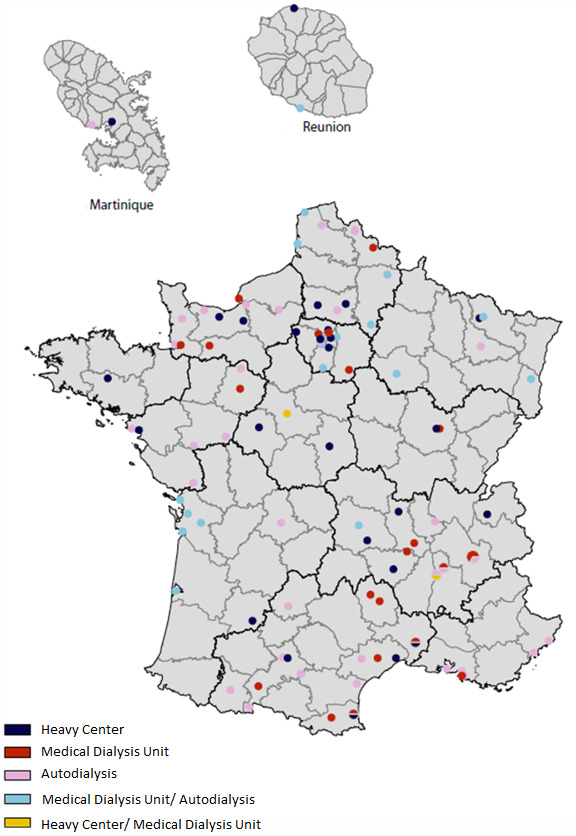
Geographic distribution of the 103 French hemodialysis units participating in the PHYDEL survey

A total of 103 hemodialysis units (14 publicly funded, 76 private non-profit, 13 private for-profit) responded to the questionnaire (response rate 51%), which corresponds to approximately 12% of all hemodialysis units in France. Figure 1 (Fig. 1) illustrates the geographic distribution of these participating units. The respondents were the hemodialysis unit’s infection control nurse (n=55), the head nurse (n=21), the infection control physician (n=8), a nephrologist (n=6), a regional care coordinator (n=5), a risk manager (n=4), the hospital director (n=3), and the Director of Nursing care (n=1). Sixty-three (61%, 95CI [52–70]) of the participating hemodialysis units reported having encountered an eHRB patient in their setting.

Table 2 (Tab. 2) describes the declared practices and protocols for identifying eHRB carriers and contacts, as well as for managing these patients, including the discontinuation of contact precautions and the varying frequencies of screening across institutions. The declared practices were very heterogeneous in all fields. As regards identification, only about one-third of HDU had implemented a process to identify at-risk patients warranting screening, and less than half of HDU reported having a computerized system to keep track of eHRB carriers and contacts. Only one-third of HDU reported screening contacts at weekly intervals as suggested by HCSP 2019 guidelines (R35) for “high risk contacts”.

In terms of management of EHRB carriers, less than one-third of HDU deployed dedicated staff as recommended by HCSP 2019 guidelines (R41) while two-thirds implemented a “march forward” strategy in which in which patients who are non-carriers of EHRB are treated first, followed by EHRB carriers; the caregivers do not return to non-carriers once they have begun treating a carrier. This strategy may be problematic in case of an emergency in a non-carrier patient, which is not unknown in these unstable patients (type and duration of precautions). The heterogeneity reported in our survey in the implementation of precautions is also retrieved regarding their discontinuation. HDU differed greatly in terms of discontinuing precautions (yes/no), and regarding the criteria for discontinuation.

Difficulties in managing eHRB were reported by 82 HDU (80%,95CI [72–87]). The reported difficulties were: the lack of standardized nationwide procedures for eHRB management in the hemodialysis setting (n=18), a delayed or inadequate alert on the eHRB status of patients (n=15), the difficulties of performing eHRB screening, the latter especially because of the reported difficulty in convincing the patient to accept the test (n=13), the difficulties related to patient transfers because of eHRB status (either those required to move to another HDU, or those prevented from being transferred to the responding HDU because of eHRB status) (n=10), the inadequate availability of single-room or single-room isolation facilities (n=9), the lack of staff knowledge regarding the management of eHRB carriers (n=7), the lack of staff (with HDU stating that substitue staff have to be competent in hemodialysis) (n=5), miscellaneous (n=10).

## Discussion

### Findings of the PHYDEL survey

The survey reveal significant heterogeneity among French HDU in their practices for identifying eHRB carriers and contact patients, as well as in managing and discontinuing contact precautions. While many units have processes in place for identifying at-risk patients, the use of computerized methods and systematic identification of eHRB contacts remains limited. Additionally, despite having protocols for managing eHRB patients and implementing precautions, there is considerable variability in practices such as screening frequency and the strategy regarding screening and contact precautions upon the return after a travel or vacations .

The study found that, in agreement with the HCSP 2019 recommendations, 37% of hemodialysis units have systems in place for identifying high-risk patients, highlighting a proactive approach in these units. No data in the literature directly address the use of such practices in other countries. This practice is valuable as it allows for the early implementation of appropriate precautions. However, it is important to note that only 41% of dialysis units utilize computerized methods for patient identification, indicating that there is room for improvement. Interestingly, the lack or delay of eHRB alerts was the second most frequent difficulty reported by HDU. Enhancing the adoption of computerized methods has the potential to significantly improve and centralize infection prevention efforts in dialysis facilities [22].

The practices for screening carriers of eHRB in hemodialysis units were highly heterogeneous, both in terms of the frequency of routine systematic screenings and certain occasions such as admissions, return after a travel or vacations, or readmissions after hospitalization. This variability in practices was reported by Marshall in 2012 [23], in care settings other than hemodialysis. More recently, similar heterogeneity in screening practices was reported by a survey in a sample of Canadian hospitals, showing that 19% and 24% of them screened dialysis patients at admission for VRE and for CPE, respectively, and 23% and 49% screen dialysis patients for VRE and for CPE, respectively, during “hospitalization” [24]. That survey dealt with different areas of the hospitals surveyed and therefore it is not clear whether the word “hospitalization” corresponds to in-patient stays of dialysis patients. However, the survey by Neitzel et al. [24] did not describe the specificities of hemodialysis units’ practices, whereas our survey provides up-to-date data. The HCSP 2019 guidelines recommend regular screening for eHRB, also in the hemodialysis setting, but does not address precisely the specific occasions of screening in hemodialysis patients (i.e., before and after holidays, which may lead the patient to go to another dialysis unit for a few days or weeks, after hospitalizations). Recommendation R55 states merely that “It is recommended to screen EHRB carriers and their contacts at regular intervals, to be defined in consultation between the hygiene team and the medical nephrology team” [18]. Given the high prevalence of EHRB in the hemodialysis setting, it would probably be helpful for hemodialysis-unit staff to have clear, standardized instructions regarding the indications and time for screening, especially as regards the recommended screening protocol to adopt when a patient under dialysis return from holidays with several dialysis sessions performed in another hemodialysis unit, or comes back to the hemodialysis unit after hospitalization in an acute or intensive care ward. It would be useful in increasing screening acceptance, since “difficulty in screening” was the third most frequent reported difficulty, partly because HDU staff find it difficult to obtain patient agreement to testing. 

Routine screening for eHRB carriers was reported by 50% of units in our survey, with 35% conducting weekly screenings, while others screened at less frequent intervals. These results are similar to those reported in Canadian hemodialysis units [24], i.e. 23% for VRE and 49% for CPE. The cost-benefit ratio of more frequent tests in hemodialysis patients has not been studied in the literature. It could be hypothesized that a more targeted approach, focusing on specific "special" occasions (e.g., return from vacation with dialysis performed in another center, etc.), might strike a better balance between cost effectiveness and IPC, while still detecting most at-risk patients. Theoretically, more frequent tests allow more timely intervention to implement contact precaution or cohorting, and possibly better control EHRB diffusion. However, the costs of tests increase automatically with their frequency, while the benefits depend both on the prevalence of eHRB in the area, on the adherence to standard precautions when the eHRB carriage has not yet be identified, on the speed of implementing contact precautions when the test comes back positive, and on the adherence to contact precautions or the feasibility of cohorting. The cost and performance of the screening test methods also play an important role. The determination of the “best” screening strategy using a decision analysis method could provide a model to help in deciding on a screening strategy for a given hemodialysis according to the relative importance of the above parameters.

The implementation of contact precautions, including dedicated personnel and cohorting (dedicated dialysis location), but also their cessation, varied widely across dialysis units in our survey. The importance of dedicating trained personnel to handle outbreaks of multidrug-resistant bacteria was confirmed from an 18-month investigation (not limited to the hemodialysis setting) conducted at a university hospital in France [25]). However, only 30% of responding units in our survey reported having dedicated personnel for managing eHRB carriers. Employing dedicated staff may be particularly difficult in a setting where nurses are highly specialized and supplementary nurses difficult to find, whether among the hospital regular staff or among temporary staff. Indeed, this difficulty was report by HDU in our survey, but only in 6^th^ position, and after the lack of suitable isolation room.

The discontinuation of contact precautions also varied widely between HDU in our survey, with some relying on repeated negative screening to cease contact precautions, where others chose to maintain long-term contact precautions. Our survey did not address the reasons of hemodialysis units for choosing either of these strategies and we were not able to assess whether this choice depended on available resources, or on the conviction of HDU managers that their staff maintained a high level of adherence to standard precautions. In the HCSP 2019 guidelines, the criteria required for stopping contact precautions, or defining the occasions where screening is necessary, indeed remain unanswered questions [18]. Here again, the heterogeneity of these practices and the lack of national recommendations suggests that more knowledge is required on these topics. 

### Implications for infection risk and screening efficacy

The choice made by each HDU regarding the screening strategy and the strategy for implementing contact precautions are of course interrelated. 

Some studies have demonstrated the benefits of frequent screening for early detection and rapid isolation with contact precautions for eHRB carriers, ultimately reducing the spread of multidrug-resistant organisms [25], [26]. However, the question of the balance between standard precautions (which should be universally applied) and contact precautions, remains controversial in the literature. Some authors suggest that well-applied standard precautions for all patients would be better than contact precautions implemented only for diagnosed eHRB carriers and suboptimal standard precautions for other patients [27]. The potential risks of misidentification and possible relaxed precautions for non-identified carriers could lead to outbreaks, undermining the overall effectiveness of infection control measures. As eHRB continue to evolve, it may be necessary to update IPC guidelines to better reflect the changing landscape of bacterial resistance and the realities of clinical practice in hemodialysis units.

## Limitations

The PHYDEL survey has several limitations. First, the response rate of 51% may introduce a selection bias, as units that chose to participate might have been more motivated or better equipped to manage infection prevention practices than those that did not respond. Additionally, the reliance on self-reported data could lead to reporting bias, with units potentially overestimating their adherence to protocols due to social desirability. Logistic challenges faced by dialysis teams in implementing recommendations may not have been fully addressed. The fact that some HDU in the survey share the same infection control teams may have led to an over representation of some answers. Finally, while the findings apply to French hemodialysis units, they may not be generalizable to other countries with differing healthcare systems and regulations. In addition, as our survey was performed just before the release of the new HCSP guidelines, we did not question HDU about the availability of a dedicated eHRB room, nor the cohorting of eHRBV carriers during the same dialysis session.

## Conclusion

This study highlights key challenges in preventing eHRB infections in French hemodialysis units, emphasizing the heterogeneity of practices among French hemodialysis units, and the need for standardized protocols, better isolation facilities, and improved communication systems. Proposed solutions include developing specific eHRB management guidelines, including screening, upgrading facility infrastructure, and implementing integrated electronic health records for streamlined information sharing, as well as a more standardized strategy across hemodialysis units in France for discontinuation of contact precautions. These efforts, coupled with ongoing staff education, aim to optimize infection prevention strategies and improve patient outcomes. Addressing these challenges proactively will help hemodialysis units mitigate eHRB transmission, ensure patient safety, and enhance overall healthcare quality for this vulnerable population.

## Notes

### Authors’ ORCIDs

- Azzi A: https://orcid.org/0000-0002-6121-1659
- Boulet L: https://orcid.org/0000-0002-5279-4290
- Vermeulin T: https://orcid.org/0000-0003-4744-3135
- Edet S: https://orcid.org/0009-0004-3645-8344
- Le Roy F: https://orcid.org/0000-0002-3818-1498
- Merle V: https://orcid.org/0000-0002-8856-7532

### Ethical approval 

The survey received approval from the local institutional review board. 

### Funding

None. 

### Acknowledgments

The authors warmly thank hemodialysis units who agreed to participate, Véronique Bellet (infection control nurse in the CoCLINNOR hygiene network), for conducting the interviews, Christine Lebaron, Marlène Legrix, Laetitia Schapman, for testing the PHYDEL questionnaire, and Dr Cécile Couchoud and Blandine Wurtz from REIN French national registry for their valuable help in data collection.

### Competing interests

The authors declare that they have no competing interests.

## Figures and Tables

**Table 1:**
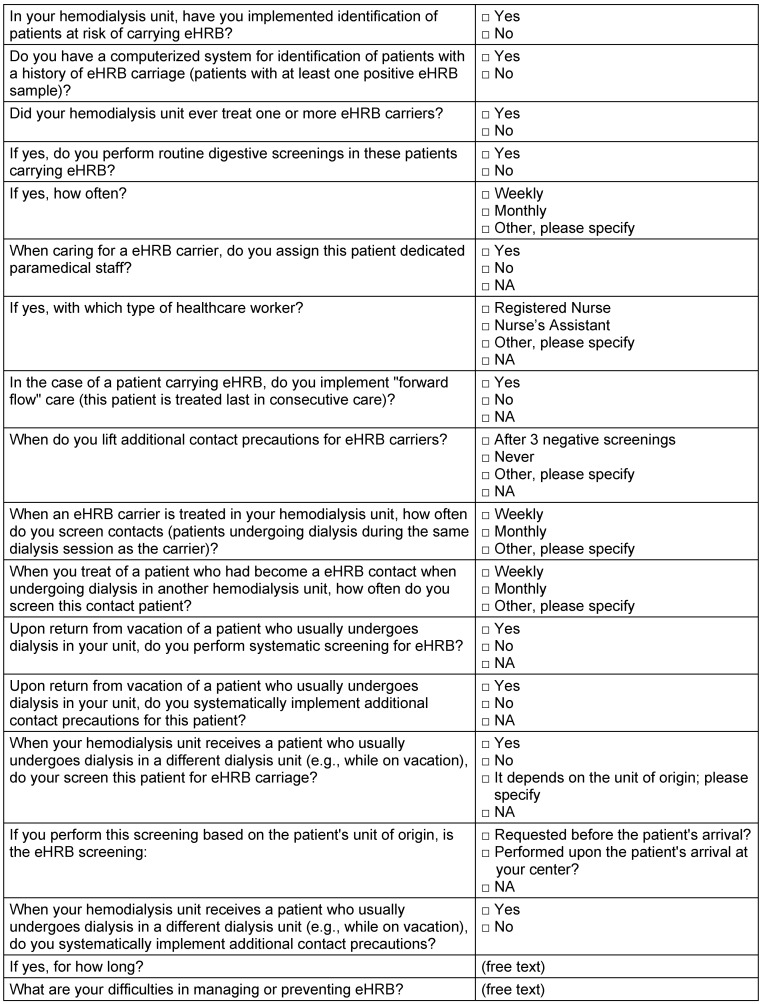
Questionnaire on practices in managing eHRB in French hemodialysis units

**Table 2:**
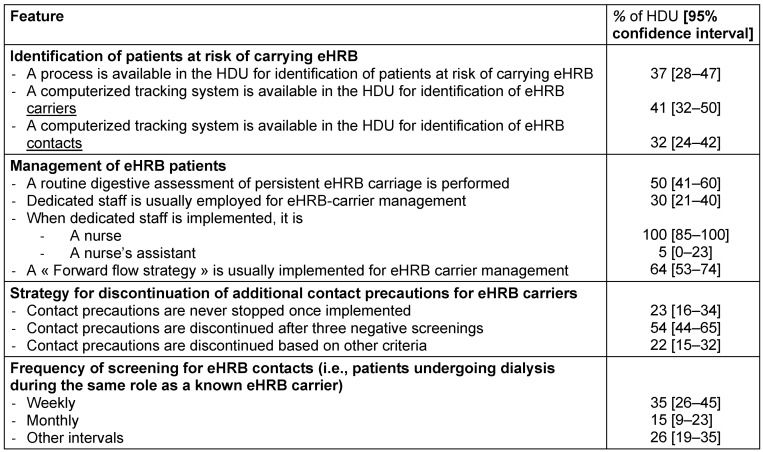
Declared processes and protocols for identifying and managing emerging highly-resistant bacteria (eHRB) patients in the 103 participating French hemodialysis units (HDU)
